# Rehabilitation and Parkinson's Disease

**DOI:** 10.1155/2012/371406

**Published:** 2012-04-05

**Authors:** Gammon M. Earhart, Terry Ellis, Alice Nieuwboer, Leland E. Dibble

**Affiliations:** ^1^Program in Physical Therapy, Department of Anatomy and Neurobiology and Department of Neurology, Washington University in St. Louis School of Medicine, St. Louis, MO 63108, USA; ^2^Department of Physical Therapy and Athletic Training, Boston University, Boston, MA 02215, USA; ^3^Department of Rehabilitation Sciences, Katholieke Universiteit Leuven, 3001 Leuven, Belgium; ^4^Department of Physical Therapy, University of Utah, Salt Lake City, UT 84108, USA

Early after the turn of the century, much excitement was generated by the reports of Tillerson et al. [1, 2] that exercise appeared to protect against neuronal degeneration in rodent models of toxin-induced parkinsonism. Such findings, coupled with epidemiologic suggestions that persons with a history of moderate to vigorous exercise may have a decreased risk of developing Parkinson's disease (PD) [3–5], led to an exponential growth in research on the effects of physical activity and exercise on PD. Unfortunately, additional follow-up animal studies to the work of Tillerson et al. have failed to yield consistent findings [6–10]. For this reason, it appears that the critical factors associated with neuroprotection remain elusive. With a continued focus on examining the effects of exercise in animal models of parkinsonism, identifying biomarkers of disease progression, and new and innovative outcomes, we look forward to a day when an evidence-based neuroprotection study can be implemented in human idiopathic PD.

 Although results from studies of the neuroprotective effects of exercise are mixed, one consistent finding from animal models and human trials is the lack of adverse effects of exercise and physical activity on anatomic and behavioral outcomes. The adverse side-effect profile of exercise as an intervention for those with PD appears to be minimal. As such, we think there is no reason to wait for confirmation of neuroprotection. Rather, evidence is accumulating that exercise and physical activity should be utilized as key tools in the management of PD across the spectrum of disease. Evidence-based approaches to rehabilitation are known to improve physical functioning, strength, balance, gait, and health-related quality of life among people with PD [11–13], but questions remain about whether or not these approaches can substantially impact fall rates [14–16]. This is a key issue, as most individuals with PD are only referred to rehabilitation after the onset of reduced mobility and an increase in falls. As such, the majority of PD rehabilitation care is provided in a tertiary prevention model of care. People with PD are most often not seen earlier in the course of the disease, when rehabilitation could play a key role in secondary preventive care. Secondary prevention would entail addressing early PD signs and symptoms, ideally immediately upon diagnosis, to optimize the condition of the central nervous system as well as other peripheral systems such as the cardiovascular and respiratory systems in order to maximize function and slow progression of disability. Even earlier intervention should be considered, as we think that rehabilitation may ultimately serve a role in primary prevention of PD. Primary prevention would entail treating those without current neurologic signs and symptoms in order to prevent PD from ever developing. Those who are potentially at risk for PD (e.g., leucine-rich repeat kinase 2 (LRRK2) carriers, those with rapid eye movement sleep behavior disorder, anosmia, constipation, abnormal positron emission tomography (PET) scans, etc.) may be excellent candidates for primary prevention.

The presently limited scope of rehabilitation in the management of PD, with utilization of rehabilitation as mainly a tertiary prevention measure, reflects a missed opportunity on the part of healthcare providers, patient advocacy groups, and patients themselves. All stakeholders in this situation should be advocates for higher expectations and should work together to develop targets for the future of rehabilitation in PD to include primary and secondary prevention and to improve our current provision of tertiary prevention interventions.

*From the Spectrum of Rehabilitation Options to Atypical Parkinsonism.* In this special issue are several articles that we hope advance the field and move us closer to these future targets. The issue opens with a series of three review articles. The first paper summarizes and synthesizes the nature and features of previous randomized, controlled trials of exercise or motor training in PD, important for the long-term provision of increasing levels of physical activity. The second paper provides a meta-analysis focused on motor learning in upper extremity tasks. The third paper provides an integrated overview of the Lee Silverman Voice Treatment approach to voice and movement therapy, discussing the rationale for the approach as well as the data regarding efficacy. These opening three articles highlight the important roles of speech, occupational, and physical therapy approaches in the rehabilitation of individuals with PD as well as address areas for future research.

Several papers in this special issue relate to gait, balance, and falls, examining the relationship between gait economy and six-minute walk distance, reviewing the literature on the costs of dual-task walking, and providing a new theoretical framework for considering freezing of gait (FOG) including methods to improve overall locomotor performance and methods to target the triggers of FOG. Also included are two randomized, controlled trials designed to improve walking. One compares visually cued walking training on a treadmill to overground walking; the other examines use of rotating treadmill training as a means of improving turning, thereby targeting a known trigger of FOG. Turning is also examined in a paper that describes turning impairments in early PD. This is followed by a paper examining the effects of medications on gait-related mobility and postural control, showing that although pharmacologic intervention enhanced some aspects of mobility, reactive postural responses did not improve. This highlights the need for awareness of postural control deficits and the need to be able to measure these deficits, as is addressed by the next two papers in the issue that present the relative merits of different balance measures across different levels of PD severity and examine the relative effectiveness of different balance measures for prospective fall prediction.

The inextricable link between posture and gait is addressed in a paper that focuses on the coupling of posture and locomotion and suggests this coupling as a specific target for rehabilitation. This link is also highlighted in two papers demonstrating that walking ability is a major contributor to fear of falling in PD, as is knee strength. The latter leads to the suggestion that resistance training may therefore be warranted as an approach to reduce fear of falling. The rationale for progressive resistance training as well as its potential mechanisms are addressed in a review paper, followed by an article examining the reliability of one repetition maximum strength testing in PD.

We conclude the issue with a set of papers addressing the delivery of evidence-based rehabilitation in different settings. These papers include two that are randomized, controlled trials of physiotherapy in outpatient and home-based settings, respectively. These are followed by a paper examining facilitators and barriers to community-based walking exercise among those with PD. Community-based healthcare for PD is also addressed in a paper describing the steps taken to improve the Dutch model of multidisciplinary care. The final paper of the issue demonstrates the effectiveness of multidisciplinary care in an inpatient setting in persons with atypical parkinsonism. All papers in this final section draw attention to the need for a collaborative, cooperative approach to rehabilitation across disciplines, across settings, and with PD and other related disorders.

Ultimately, we believe that there is a need to redefine the role of rehabilitation in PD to include the provision of primary, secondary, and tertiary prevention approaches. Across this spectrum from primary through tertiary care, the application of multidisciplinary approaches is needed to optimize the health, function, and quality of life of individuals at risk for, or who already have, PD. Only then will the full potential of rehabilitation in the management of PD be realized.

*Gammon M. Earhart* *Gammon M. Earhart* *Terry Ellis* *Terry Ellis* *Alice Nieuwboer* *Alice Nieuwboer* *Leland E. Dibble* *Leland E. Dibble*
